# Genome Sequence of *Elizabethkingia anophelis* Strain EaAs1, Isolated from the Asian Malaria Mosquito *Anopheles stephensi*

**DOI:** 10.1128/genomeA.00084-16

**Published:** 2016-03-10

**Authors:** Juan Antonio Raygoza Garay, Grant L. Hughes, Vikas Koundal, Jason L. Rasgon, Michael M. Mwangi

**Affiliations:** a Department of Biochemistry and Molecular Biology, Center for Infectious Disease Dynamics, Pennsylvania State University, University Park, Pennsylvania, USA; b Huck Institutes of the Life Sciences, Pennsylvania State University, University Park, Pennsylvania, USA; c Department of Pathology and Institute for Human Infections and Immunity, University of Texas Medical Branch, Galveston, Texas, USA; d Department of Entomology, Center for Infectious Disease Dynamics, Pennsylvania State University, University Park, Pennsylvania, USA

## Abstract

We sequenced the genome of a strain of the Gram-negative bacterial species *Elizabethkingia anophelis*, which is an important component of the *Anopheles* mosquito microbiome. This genome sequence will add to the list of resources used to examine host-microbe interactions in mosquitoes.

## GENOME ANNOUNCEMENT

*Anopheles* mosquitoes possess a diverse bacterial microbiome, of which the Gram-negative genus *Flavobacterium* is a major component (1, 2). *Elizabethkingia anophelis* is a member of the *Flavobacteriaceae*, and recently, several *E. anophelis* strains from the African malaria vector *Anopheles gambiae* have had their genomes sequenced (3, 4). Here, we report the genome sequence of an *E. anophelis* strain from the Asian malaria vector *Anopheles stephensi*.

A homogenate of surface-sterilized *A. stephensi* (Liston strain) was used to inoculate LB agar plates, and a single colony was isolated and confirmed by 16S rRNA gene sequencing to be *E. anophelis*. Genomic DNA was extracted using a Qiagen blood and tissue kit, according to the recommendations for bacteria, and sequenced in a 500-cycle run on an Illumina MiSeq at the Pennsylvania State University Genomics Core Facility, University Park, PA. The DNA library was prepared using a Nextera XT DNA library preparation kit, with an insert size of 400 bp. The 250-bp paired-end reads were assembled using MIRA version 4.0 and the assembly refined using DNAStar SeqMan Pro version 12.0, resulting in a total of 12 contigs with a combined length of 3.6 Mbp, an *N*_50_ of 491,796 bp, a median read coverage of 70×, and an average G+C content of 36%. The annotation was done using the RAST pipeline (5–7), followed by manual curation, yielding 3,324 protein-coding genes (CDSs) and 42 RNA genes.

Ninety-five genes with homology to proteins involved in antimicrobial and toxin resistance were identified, including 22, 47, and 16 genes with homology to heavy-metal resistance, multidrug efflux, and β-lactam resistance proteins, respectively. Of interest in using this organism as a tool for vector studies, genes conferring resistance to tetracycline [*tet*(A)], chloramphenicol, macrolides, and acriflavine are present. Interestingly, a gene with high homology to *vanW* is present. VanW has unknown function but has been assumed to be related to vancomycin resistance, since it has been found in the *vanB* and *vanG* gene clusters in Gram-positive bacteria (8, 9). However, Gram-negative bacteria (except nongonococcal *Neisseria* species) do not need vancomycin resistance genes, since they are intrinsically resistant to the antibiotic (10), suggesting that closely related homologs of *vanW*, and perhaps *vanW* itself, have functions that are independent of vancomycin resistance.

Native mosquito microbes have the innate ability to interfere with pathogen transmission (11, 12) and can be manipulated to express antipathogen effector molecules (13). A recent report demonstrates that *E. anophelis* is amenable to transformation and is present in all mosquito life stages (14), opening the possibility of using this bacterium in vector control. However, concerns about pathogenicity need to be addressed if this bacterium is to be used in an applied manner. The sequencing of this bacterial isolate of *E. anophelis* may help clarify the role of *E. anophelis* as an emerging human pathogen (15) and adds to the resources of bacterial sequences (16, 17) for studying host-microbiome interactions in the Asian malaria vector *A. stephensi*.

### Nucleotide sequence accession number.

This whole-genome shotgun project has been deposited at GenBank under the accession no. LFKT00000000.
